# Clusterin deficiency exacerbates cholestatic liver disease through ER stress and NLRP3 inflammasome activation

**DOI:** 10.1186/s13578-025-01376-z

**Published:** 2025-03-15

**Authors:** Hye-Young Seo, Ji Yeon Park, So-Hee Lee, Hye Won Lee, Eugene Han, Jae Seok Hwang, Mi Kyung Kim, Byoung Kuk Jang

**Affiliations:** 1 Department of Internal Medicine, School of Medicine, Institute for Medical Science, Keimyung University, Daegu, Korea; 2 Department of Pathology, Keimyung University School of Medicine, Daegu, Korea

**Keywords:** Clusterin, Cholestatic liver, ER stress, NLRP3 inflammasome

## Abstract

**Background:**

Cholestatic liver disease, characterized by impaired bile flow, leads to the accumulation of harmful metabolites and toxins, resulting in liver damage. Inflammatory cytokines are crucial for the progression of this condition. Clusterin is a glycoprotein with roles in cell death, lipid transport, and cellular protection. We previously demonstrated that clusterin protects against hepatic steatosis and hepatic fibrosis. This study explored the roles of clusterin in cholestatic liver injury induced by a DDC (3,5-diethoxycarbonyl-1,4-dihydrocollidine) diet.

**Methods:**

The study evaluated the impact of clusterin on liver injury in C57BL/6 mice and clusterin-knockout (KO) mice fed a DDC diet for 10–20 days. Primary Kupffer cells (KCs) and hepatocytes (HCs) of these mice were analyzed. Techniques such as Sirius red staining, immunohistochemistry, real-time RT-PCR, enzyme-linked immunosorbent assays, and western blotting were performed to assess the effects of clusterin.

**Results:**

Clusterin expression was upregulated in the cholestatic liver. Clusterin-KO mice exhibited elevated levels of alanine aminotransferase, aspartate aminotransferase, collagen, and αSMA upon DDC diet-induced liver injury. They also had increased levels of markers of endoplasmic reticulum (ER) stress (CHOP, ATF6, and p-eIF2α) and inflammasome activity (NLRP3, ASC, caspase-1, and interleukin 1 beta (IL1β) protein expression, and IL1β and interleukin 18 secretion). Thapsigargin, an ER stress inducer, heightened NLRP3 inflammasome activation in primary KCs and HCs, which was mitigated by overexpression of clusterin.

**Conclusions:**

The absence of clusterin exacerbates ER stress and NLRP3 inflammasome activation in mice fed a DDC diet. Conversely, overexpression of clusterin suppresses these stress responses. Thus, clusterin deficiency is associated with an enhanced inflammasome response in the liver that is linked to upregulation of ER stress.

**Supplementary Information:**

The online version contains supplementary material available at 10.1186/s13578-025-01376-z.

## Introduction

Cholestatic liver disease, including primary biliary cholangitis (PBC), is marked by impaired bile flow, leading to the buildup of bile acids and toxins in the liver and bloodstream. This accumulation results in significant liver injury [1]. Inflammation significantly contributes to the progression of liver injury and fibrosis in cholestasis. Recent studies have highlighted the critical role of inflammatory cytokines in the development of cholestatic liver injury, which is characterized by chronic bile duct inflammation [2–4].

The endoplasmic reticulum (ER) is an important organelle responsible for protein synthesis, transport, folding, and modification. Several studies reported that ER stress contributes to the development of liver disease [5], and recent studies showed that cholestasis triggers an ER stress response [6, 7]. Additionally, ER stress can cause liver injury by affecting the NLRP3 inflammasome in hepatocytes (HCs) [8–10]. The NLRP3 inflammasome is a cytoplasmic multiprotein complex expressed not only in innate immune cells like macrophages but also in non-immune cells such as HCs and hepatic stellate cells (HSCs) [11]. It has been implicated in the pathogenesis of liver conditions like non-alcoholic fatty liver disease, steatohepatitis, and fibrosis [12–14]. Given this role, both ER stress and the NLRP3 inflammasome are currently being explored as vital targets to develop treatments for liver diseases.

Clusterin, also known as apolipoprotein J, is a secretory glycoprotein and heterodimeric multifunctional chaperone molecule [15]. It is expressed in a variety of tissues and body fluids, and its expression levels are significantly increased in various pathological conditions [16]. Clusterin has been reported to protect against insulin resistance and inflammation by regulating signaling pathways such as the phosphoinositide 3-kinase and NF-κB pathways, particularly in macrophages [17–20]. A recent study reported that clusterin attenuates inflammation induced by cholesterol crystals by inhibiting the NLRP3 inflammasome pathway in THP-1 macrophages [21]. These results suggest that clusterin also plays an important role in regulating inflammation-related conditions; however, few studies have investigated its role in regulating bile acid-induced liver injury. In this study, we elucidated the mechanisms by which clusterin protects against cholestatic liver injury.

## Materials and methods

### Materials

Thapsigargin (Tg, *Escherichia coli* 055, B5) and an anti-αSMA (A2547) antibody were purchased from Sigma-Aldrich (St. Louis, MO, USA). Antibodies against clusterin (SC6420), CHOP (SC7351), ATF6 (SC166659), CTGF (SC365970) and ASC (SC514414) were purchased from Santa Cruz Biotechnology (Dallas, TX, USA). Antibodies against interleukin 1 beta (IL1β, ab9722) and gasdermin D (GSDMD, ab219800) were purchased from Abcam (Cambridge, UK). Antibodies against collagen (PA529569) and T-eIF2α (AH00802) were purchased from Thermo Fisher Scientific (Waltham, MA, USA). Antibodies against NLRP3 (CS1510), cleaved caspase 1 (CS89332), GAPDH (CS2118), tubulin (CS2146), caspase 1 (CS2225), cleaved caspase 1 (CS89332), p- eIF2α (CS9721), BIP (CS3183), p-STAT3 (CS9138), and STAT3 (CS4904), as well as anti-rabbit (7074P2) and anti-mouse (7076P2) secondary antibodies, were purchased from Cell Signaling Technology (Beverly, MA, USA).

### Animals and diets

In vivo experiments were conducted using 8-week-old male C57BL/6 mice (Central Lab Animal, Seoul, Korea). To generate clusterin-knockout (KO) mice on the C57BL/6 genetic background, clusterin-deficient mice that were originally generated using a Black Swiss genetic background were backcrossed with the C57BL/6 strain for at least seven generations. All experiments were approved by the Institutional Animal Care and Use Committee of Keimyung University (KM-2023-32). All animal procedures were carried out in accordance with institutional guidelines for animal research. C57BL/6 and clusterin-KO mice were fed a DDC (3,5-diethioxycarbonyl-1,4-dihydrocollidine) diet (0.1% wt/wt) for 10 or 20 days and then sacrificed (*n* = 4 per group). Control animals were fed a control diet. The liver and blood were collected for subsequent analyses.

### Bile duct ligation (BDL)

Mice were assigned to four groups: sham-operated wild-type (WT) mice (*n* = 2), sham-operated clusterin-KO mice (*n* = 2), WT mice subjected to BDL for 3 days (*n* = 2), and clusterin-KO mice subjected to BDL for 3 days (*n* = 3). Animals in the BDL groups were anesthetized with pentobarbital (50 mg/kg) and underwent a midline laparotomy. The common bile duct was doubly ligated with 5 − 0 silk and then transected between the ligatures. For sham-operated mice, the procedure was identical except the ligation step was omitted.

### Patients and specimens

Liver tissues were obtained from 12 patients with PBC who underwent biopsy at Keimyung University, Dongsan Medical Center, between January 2002 and December 2018. Six normal adjacent liver tissues biopsied for non-inflammatory or neoplastic diseases were used as a control. The study was conducted in accordance with the Declaration of Helsinki, and the protocol was reviewed and approved by the Institutional Review Board of Keimyung University Dongsan Hospital (IRB No. 2023-11-020). Immunohistochemical staining was performed using an anti-clusterin antibody (Santa Cruz Biotechnology) and an automatic staining device (Benchmark XT; Ventana Medical Systems, Mountain View, CA, USA) in accordance with the manufacturers’ protocols.

### Immunohistochemical analysis

Livers were isolated from mice, fixed in 4% formaldehyde, and then paraffin-embedded. Histochemical staining was performed using hematoxylin and eosin and Sirius red. Immunohistochemical staining was performed by incubation with primary antibodies against clusterin (1:200), NLRP3 (1:200), IL1β (1:200), ATF6 (1:200) and CHOP (1:200) followed by horseradish peroxidase-conjugated anti-rabbit and anti-mouse (Dako, Glostrup, Denmark), and anti-goat (Abcam) IgG secondary antibodies. All data were normalized against the equivalent data in mice fed chow (control).

### Isolation of primary Kupffer cells (KCs) and hepatocytes (HCs)

All experiments were approved by the Institutional Animal Care and Use Committee of Keimyung University (KM-2023-32). All animal procedures were performed in strict accordance with institutional guidelines for animal research. All surgeries were performed under sodium pentobarbital anesthesia, and every effort was made to minimize pain. Mouse KCs and HCs were obtained by perfusion of EGTA solution and collagenase solution (collagenase type I; Worthington Biochemical Corp., Lakewood, NJ, USA) through the portal vein of C57BL/6 mice. The liver was then incubated at 37 °C for 20 min, filtered through a 70 μm nylon mesh, and centrifuged at 500 rpm for 5 min to separate the HC pellet and supernatant containing KCs. HCs were resuspended in Williams’ medium E (Sigma-Aldrich), cultured in type I collagen-coated dishes (IWAKI Scitech Kiv, Tokyo, Japan) for 1–2 h, and then cultured with medium 199 (Sigma-Aldrich). The HC pellet and separated supernatant were centrifuged at 1600 rpm for 10 min, and then the pellet was subjected to OptiPrep (Sigma-Aldrich) density-gradient centrifugation to isolate KCs. The isolated KCs were plated in RPMI 1640 medium (Gibco-BRL, Grand Island, NY, USA) containing 10% fetal bovine serum (FBS) and incubated for 30 min. The medium was then replaced to obtain purified KCs. KCs and HCs were pretreated with compounds in the presence of 0.5% FBS with or without adenovirus clusterin infection for 2 h and then treated with Tg for 24 h.

### Isolation of primary hepatic stellate cells (HSCs)

HSCs were isolated from WT C57BL/6 and clusterin-KO mice by perfusing the liver through the inferior vena cava. The liver was perfused with EGTA buffer (136.89 mmol/L NaCl, 5.37 mmol/L KCl, 0.64 mmol/L NaH2PO4.H2O, 0.85 mmol/L Na2HPO4, 9.99 mmol/L HEPES, 4.17 mmol/L NaHCO3, 0.5 mmol/L EGTA, and 5 mmol/L glucose [pH 7.35–7.4]) at a rate of 5 mL/min for 2 min, followed by Enzyme buffer (136.89 mmol/L NaCl, 5.37 mmol/L KCl, 0.64 mmol/L NaH2PO4.H2O, 0.85 mmol/L Na2HPO4, 9.99 mmol/L HEPES, 4.17 mmol/L NaHCO3, and 3.81 mmol/L CaCl2.2H2O [pH 7.35–7.4]) containing 0.4 mg/mL pronase (Roche Diagnostics, Indianapolis, IN, USA) at a rate of 5 mL/min for 5 min and then Enzyme buffer containing 0.193 U/mg collagenase (Roche Diagnostics) at a rate of 5 mL/min for 7 min. After perfusion, the liver was shaken for 25 min at 37 ℃, filtered through a 70 μm nylon mesh, and centrifuged at 580 × g for 10 min at 4 ℃. Pelleted HSCs were resuspended in Gey’s Balanced Salt Solution (GBSS, Sigma-Aldrich), gently overlaid with a gradient of Cell-OptiPrep™ (Sigma-Aldrich) prepared with GBSS using a pipette, and then centrifuged at 1380 × g for 17 min at 4 °C without braking. HSCs present in a thin white layer at the interface between Cell-OptiPrep™ and GBSS were harvested and washed with Hank’s Balanced Salt Solution. The cells were plated in DMEM (Gibco-BRL) containing 10% FBS, and the medium was changed every 2 days.

### Cell culture

The human monocytic cell line THP-1 (Korean Cell Line Bank, Seoul, Korea) and the human HSC line LX2 were cultured in DMEM (Gibco-BRL) supplemented with 10% FBS (Hyclone, Logan, UT, USA) and antibiotics in 5% CO_2_/95% air at 37 °C. The cells were serum-starved in medium containing 0.5% FBS and then treated as indicated in the main text.

### Generation of a recombinant adenovirus

cDNA encoding rat clusterin was inserted into the pAd-Track-CMV shuttle vector. To produce the recombinant adenoviral plasmid, the resultant shuttle vector was electroporated into BJ5138 cells containing the AdEasy adenoviral vector. Recombinant adenoviral plasmids were transfected, and adenoviruses expressing clusterin were amplified in human embryonic kidney-293 cells and purified using CsCl density centrifugation (Sigma-Aldrich). The viruses were collected and desalted, and the titers were determined using an Adeno-X Rapid Titer Kit (BD Bioscience, San Jose, CA, USA).

### Quantitative real-time RT-PCR

Total RNA was extracted using TRIzol reagent (Life Technologies, Grand Island, NY, USA). Reverse transcription was performed using a Maxima First Strand cDNA Synthesis Kit (Thermo Scientific, Rockford, IL, USA). Quantitative real-time RT-PCR was performed using SYBR Green Master Mix (Roche Diagnostics) and the CFX Connect Real-Time PCR System (Bio-Rad, Richmond, CA, USA). The primer sequences are listed in Supplementary Table 1.

### Western blotting

Liver tissue and cells were lysed with RIPA lysis buffer (Thermo Fisher Scientific) supplemented with a cocktail of protease/phosphatase inhibitors (genDEPOT, Katy, TX, USA). Proteins were separated by SDS-PAGE and then electrophoretically transferred to a polyvinyl difluoride membrane (Millipore, Burlington, MA, USA). The membranes were sequentially incubated with primary antibodies and appropriate horseradish peroxidase-conjugated secondary antibodies. Signals were visualized using a Clarity™ Western ECL Substrate Kit (Bio-Rad). Signal intensities were quantitated by densitometry using ImageJ software (version 1.52a) (NIH, Bethesda, MD, USA), with protein bands normalized to GAPDH for quantitative comparisons.

### Measurement of cytokine levels

Mouse serum and cell culture medium were stored at − 80 °C. Levels of IL1β and interleukin 18 (IL18) were measured using enzyme-linked immunosorbent assay (ELISA) kits (R&D Systems, Abingdon, UK), following the manufacturer’s instructions.

### Statistical analysis

Experimental results were statistically analyzed by a one-way analysis of variance with the Bonferroni correction or the two-tailed Student’s t test (GraphPad, Prism version 9.5.1). *P* < 0.05, *P* < 0.01 and *P* < 0.001 were considered statistically significant. Data are presented as mean ± standard error of the mean (SEM). All experiments were performed at least three times.

## Results

### Expression of clusterin is increased in the cholestatic liver

**Fig. 1:**
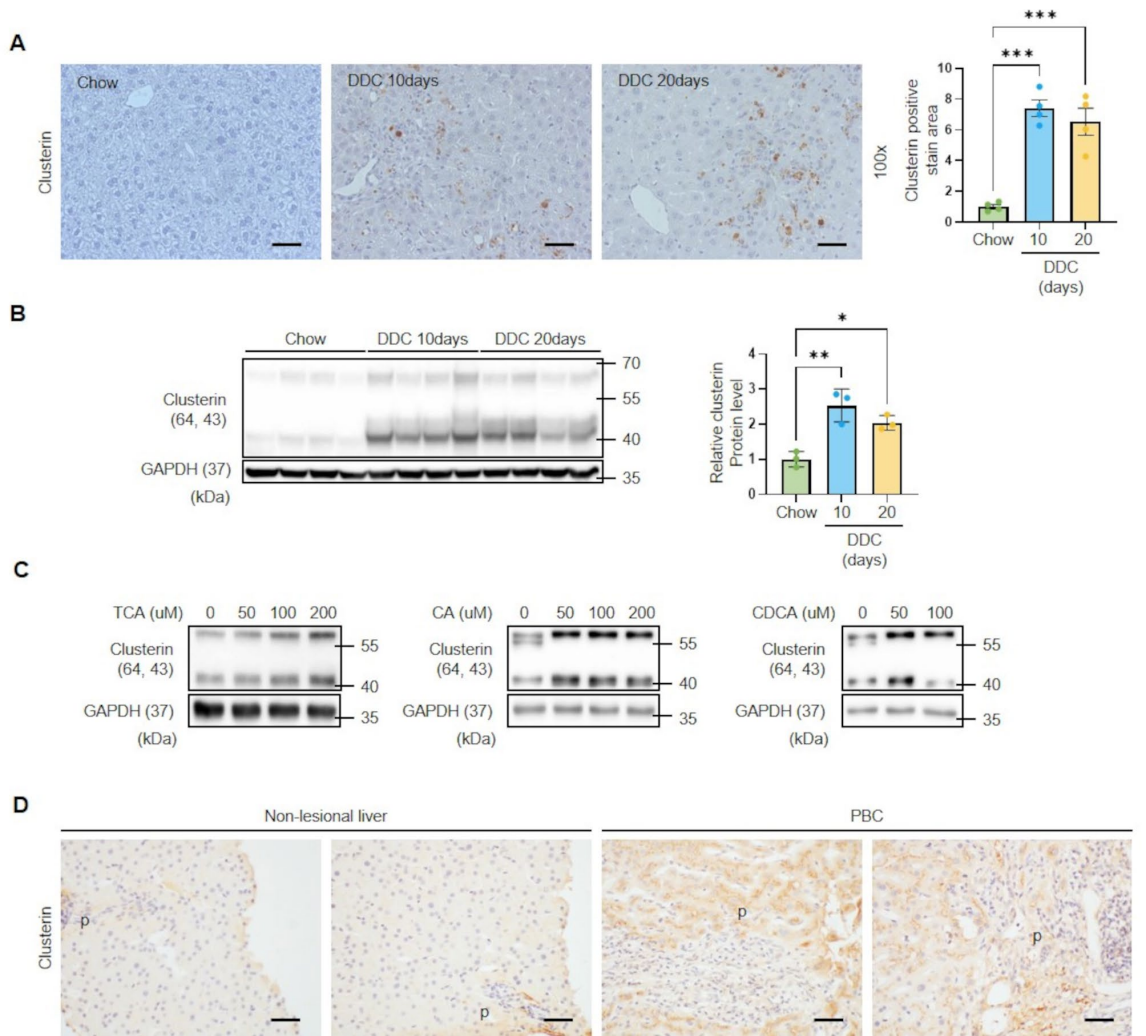
Clusterin expression in response to a DDC diet and bile acids. (**A**) Representative immunohistochemical images of clusterin expression in liver tissues of C57BL/6 mice fed a DDC diet for 10 (*n* = 4) and 20 days (*n* = 4) compared with controls (*n* = 4). Data in the bar graph are means ± SEM. ***p < 0.001. Scale bars indicate 100 μm. (**B**) Representative western blot analysis of clusterin expression in liver tissues of mice fed a DDC diet. Data in the bar graph are means ± SEM. *p < 0.05, **p < 0.01. (**C**) Representative western blot analysis of clusterin expression in lysates of primary HCs treated with TCA, CA, and CDCA. (**D**) Immunohistochemical analysis of clusterin in liver sections from patients with PBC compared with control liver tissues. Scale bars indicate 100 μm. p: portal tract

First, we investigated whether the expression level of clusterin is altered in cholestatic liver disease model. To investigate the potential involvement of clusterin in cholestatic liver injury, we employed mice fed a DDC (3,5-diethoxycarbonyl-1,4-dihydrocollidine) diet as an experimental model. Immunohistochemical (IH) analyses of liver tissues after feeding C57BL/6 a DDC diet for 10 and 20 days, showed clusterin expression exhibited elevation compared to control mice (Fig. 1A). Protein expression of liver tissue in DDC diet mice were also increased compared to those of control mice (Fig. 1B). The DDC diet causes liver injury due to cholestasis, which lead to bile acid accumulation in the liver. Therefore, we treated primary hepatocytes with the bile acids TCA, CDCA and CA and confirmed the expression of clusterin (Fig. 1C), and when we further studied the time course of clusterin expression by CA and CDCA (100 μM), both significantly increased clusterin expression in a time dependent manner (Figrue S1A). Clusterin was also elevated in liver sections from patients with primary biliary cholangitis, which showed an enhanced peritubular pattern in zone 1 hepatocytes and weak cytoplasmic expression in nonlesional parenchymal hepatocytes in patients with PBC (Fig. 1E). However, DDC fed mice and bile acids had no effect on the mRNA expression of clusterin (Figure S1B, C). These results suggest that clusterin is post-transcriptionally regulated by bile acids.

### Loss of clusterin accelerates DDC diet-induced liver injury

**Fig. 2:**
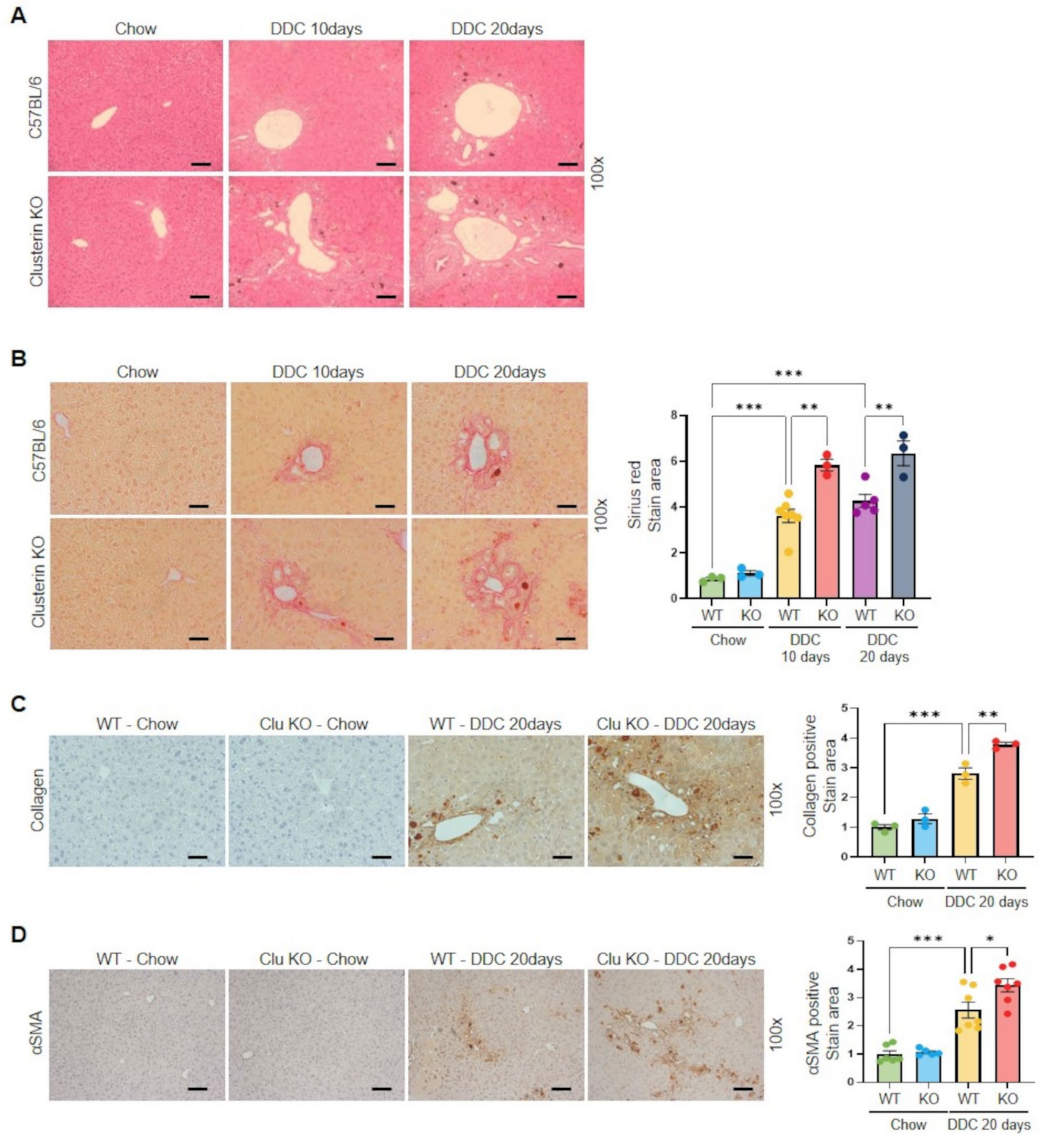
Clusterin deficiency promotes fibrosis upon DDC diet-induced liver injury. (**A**,** B**) Representative images of hematoxylin and eosin (**A**) and Sirius red (**B**) staining in WT and clusterin-KO mice fed a DDC diet (*n* = 4). **p < 0.01, ***p < 0.001. (**C**,** D**) Immunohistochemical analysis of collagen (**C**) and αSMA (**D)** expression in WT and clusterin-KO mice fed a DDC diet. Data in the bar graph are means ± SEM. *p < 0.05, **p < 0.01, ***p < 0.001. Scale bars indicate 100 μm

**Fig. 3:**
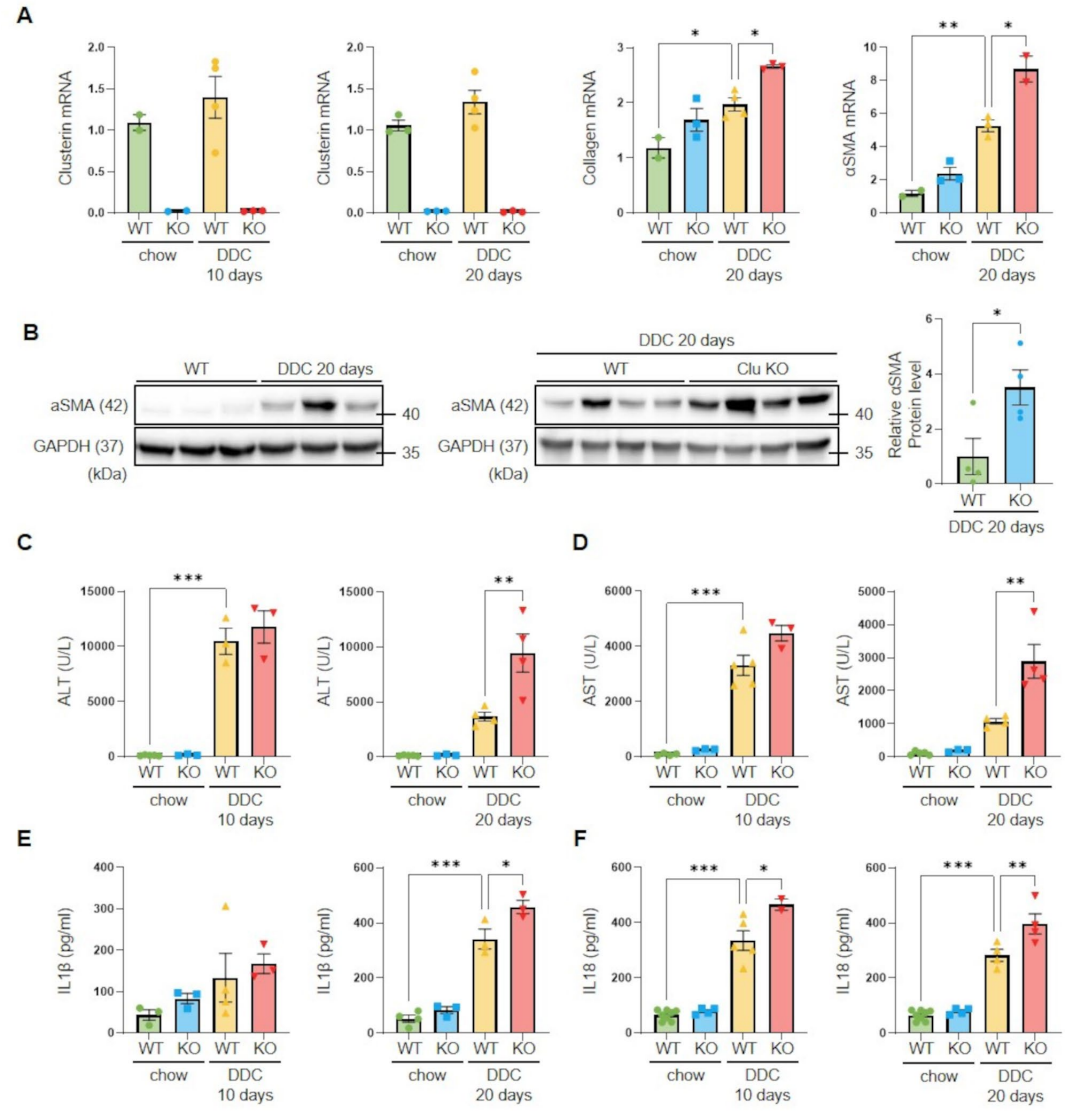
Effects of clusterin deficiency on DDC diet-induced liver injury. (**A**,** B**) Representative real-time RT-PCR (**A**) and western blot (**B**) analyses of type I collagen and αSMA expression in WT and clusterin-KO mice fed a DDC diet for 20 days. *p < 0.05, **p < 0.01. (**C**,** D**) ELISAs of ALT (**C**) and AST (**D**) levels in serum of WT and clusterin-KO mice fed a DDC diet for 10 and 20 days. (**E**,** F**) ELISAs of IL1β (**E**) and IL18 (**F**) levels in serum of WT and clusterin-KO mice fed a DDC diet for 10 and 20 days. *p < 0.05, **p < 0.01, ***p < 0.001

Next, to determine the effect of clusterin on cholestatic liver disease, clusterin-KO mice were fed a DDC diet for 10 or 20 days. Hematoxylin and eosin staining detected significant structural distortion of lobular structures in mice with DDC-induced liver injury, which was further exacerbated in clusterin-KO mice (Fig. 2A). Sirius red staining revealed that fibrosis was more severe in clusterin-KO mice than in WT mice fed a DDC diet (Fig. 2B). Immunohistochemical staining showed that expression of collagen and αSMA was higher in clusterin-KO mice fed a DDC diet for 20 days (Fig. 2C, D). In addition, real-time RT-PCR and western blot analyses revealed that expression of type I collagen and αSMA was higher in clusterin-KO mice fed a DDC diet for 20 days than in WT mice fed a DDC diet for 20 days (Fig. 3A, B). Serum alanine aminotransferase (ALT) and aspartate aminotransferase (AST) levels were also significantly higher in DDC-fed mice than in controls, and were further increased in clusterin-KO mice fed a DDC diet for 20 days (Fig. 3C, D). In addition, serum levels of the pro-inflammatory cytokines IL1β and IL18 were increased in DDC-fed mice and were significantly higher in clusterin-KO mice fed a DDC diet than in WT mice fed a DDC diet (Fig. 3E, F). We previously reported that clusterin expression is increased in a BDL model [25]. Building on this finding, we further investigated the role of clusterin upon BDL. Trichrome staining and αSMA protein expression analysis revealed that fibrosis was significantly more severe in clusterin-KO mice subjected to BDL than in WT mice subjected to BDL (Fig. S2A, B). Serum analysis indicated the levels of ALT, AST, and IL1β were elevated in clusterin-KO mice subjected to BDL (Fig. S2C, D), indicative of more severe liver damage and inflammation. These results emphasize the crucial role of clusterin in modulating responses to liver disease and highlight its potential as a therapeutic target to manage cholestatic liver diseases characterized by fibrosis.

### Clusterin deficiency promotes NLRP3 inflammasome activation upon DDC diet-induced liver injury

**Fig. 4:**
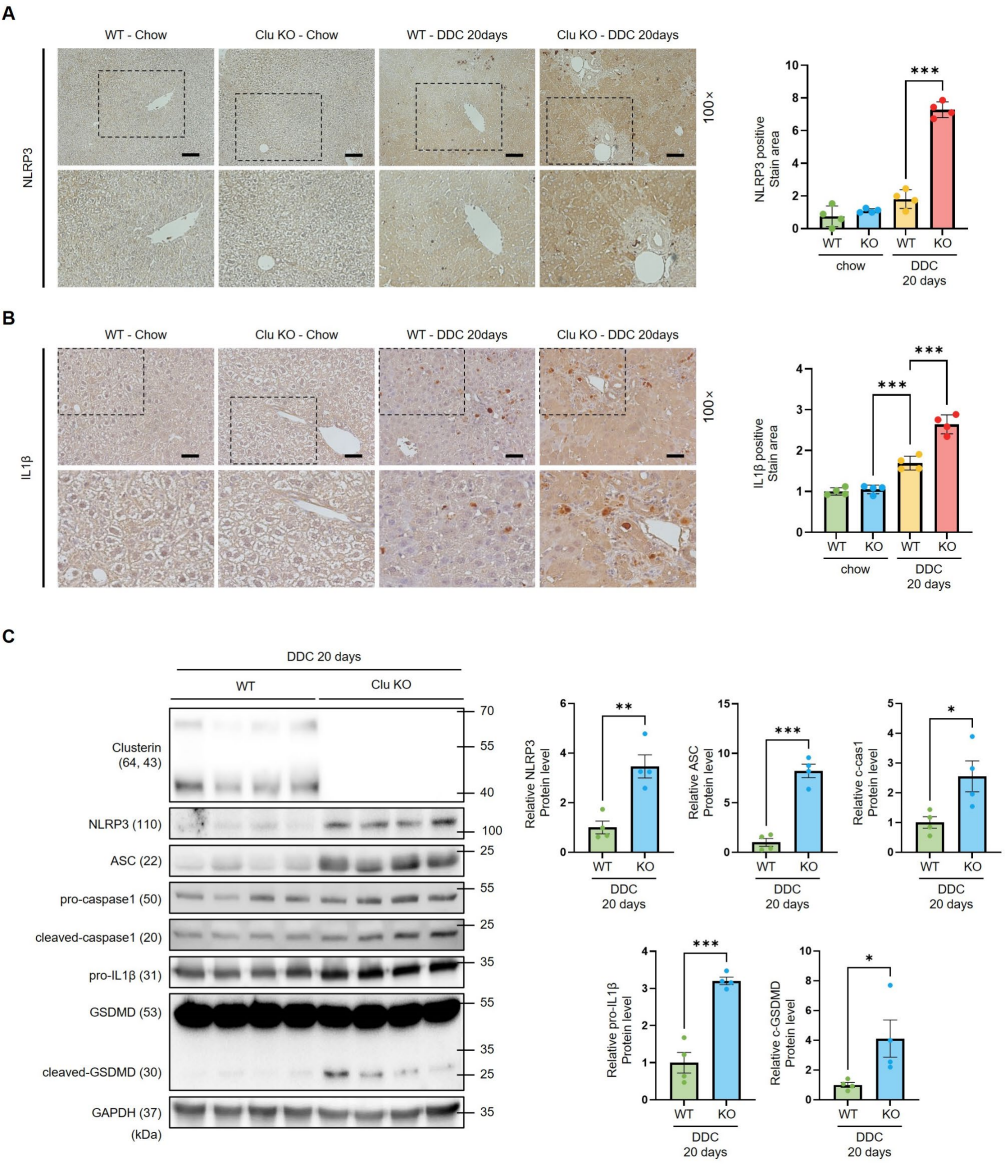
NLRP3 inflammasome activation in clusterin-KO mice fed a DDC diet. (**A**,** B**) Immunohistochemical analysis of NLRP3 (**A**) and IL1β (**B**) expression in WT and clusterin-KO mice fed a DDC diet for 20 days. Data in the bar graph are means ± SEM. ***p < 0.001. Scale bars indicate 100 μm. (**C**) Representative western blot analysis of NLRP3 inflammasome markers in WT and clusterin-KO mice fed a DDC diet for 20 days. *p < 0.05, **p < 0.01, ***p < 0.001

Inflammasomes are reportedly activated in the livers of cholestatic patients, and upregulation of IL1β and IL18 is associated with inflammasomes; therefore, we investigated whether loss of clusterin in mice fed a DDC diet is associated with NLPR3 inflammasome activation. Immunohistochemical staining showed that expression of NLRP3 and IL1β was increased in clusterin-KO mice fed a DDC diet for 20 days (Fig. 4A, B). In addition, western blot analysis showed that protein expression of the NLRP3 inflammasome markers NLRP3, ASC, caspase-1, IL1β, and GSDMD was significantly higher in clusterin-KO mice fed a DDC diet for 20 days than in WT mice fed a DDC diet for 20 days (Fig. 4C). Protein expression of NLRP3 was also higher in clusterin-KO mice subjected to BDL than in WT mice subjected to BDL (Fig. S2E). These results suggest that clusterin deficiency promotes NLRP3 inflammasome activation during cholestasis and exacerbates NLRP3 inflammasome activation upon BDL, highlighting the key role of clusterin in modulating liver disease responses in different models of liver injury.

### Clusterin deficiency enhances ER stress-induced NLRP3 inflammasome activation

**Fig. 5:**
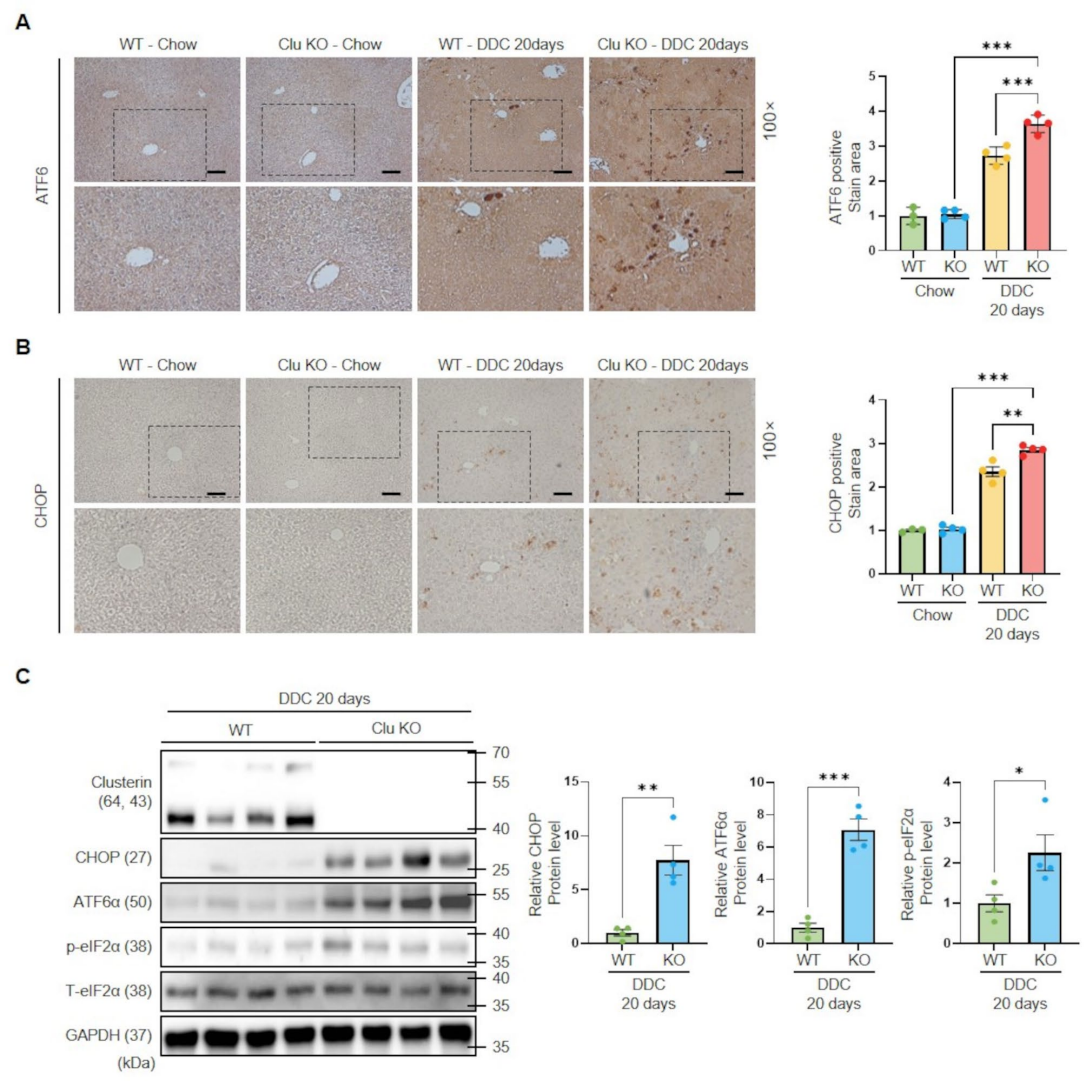
ER stress response in clusterin-KO mice fed a DDC diet. (**A**,** B**) Immunohistochemical analysis of ATF6 (**A**) and CHOP (**B**) expression in WT and clusterin-KO mice fed a DDC diet for 20 days. Data in the bar graph are means ± SEM. **p < 0.01, ***p < 0.001. Scale bars indicate 100 μm. (**C**) Representative western blot analysis of ER stress markers in WT and clusterin-KO mice fed a DDC diet for 20 days. *p < 0.05, **p < 0.01, ***p < 0.001

**Fig. 6:**
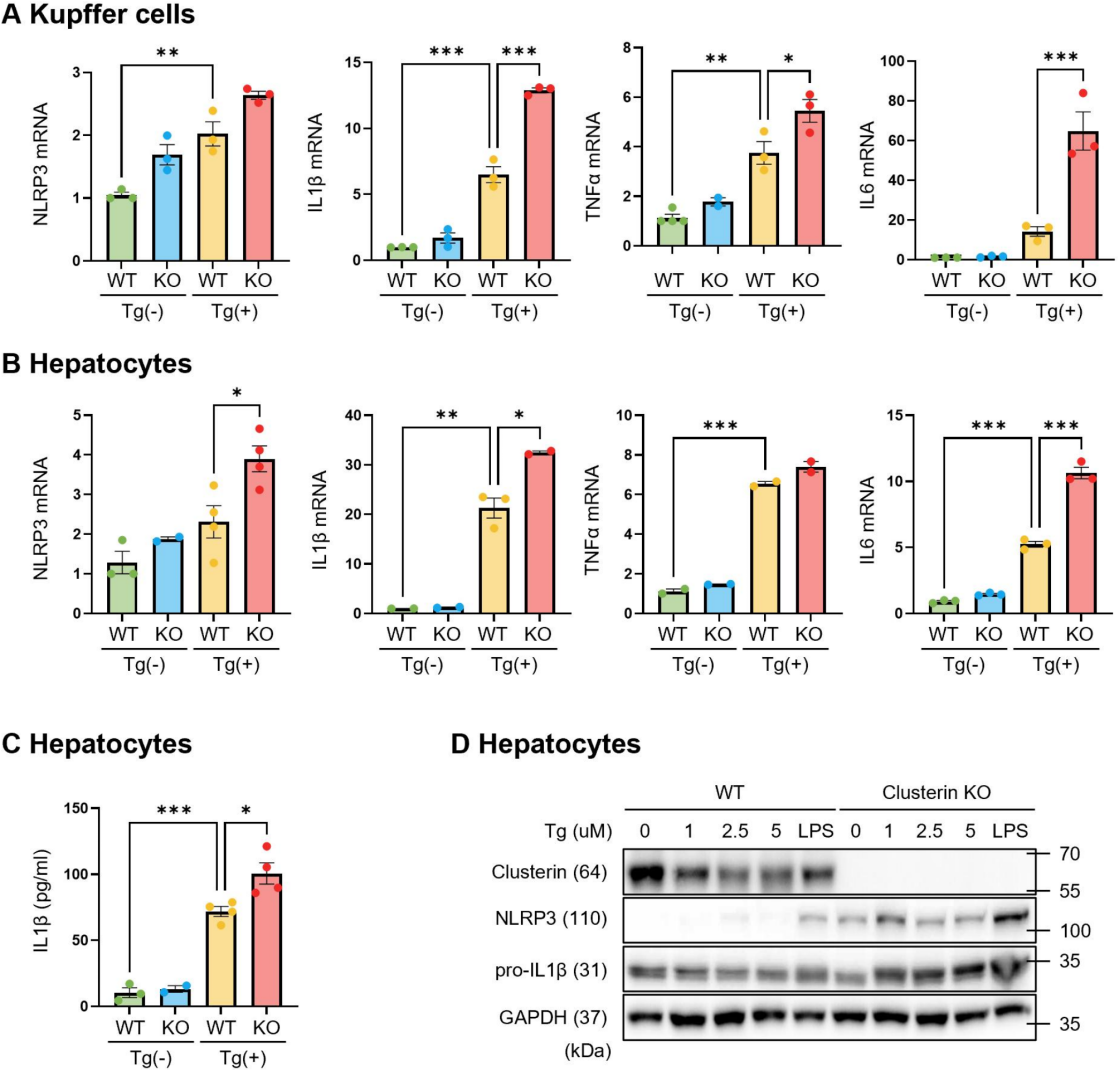
ER stress enhances NLRP3 inflammasome activation in clusterin-KO primary HCs and KCs (**A, B**) Representative real-time RT-PCR analysis of NLRP3, IL1β, TNFα, and IL6 mRNA levels in clusterin-KO primary KCs (**A**) and HCs (**B**) treated with Tg (1 μM, 24 h). *p < 0.05, **p < 0.01, ***p < 0.001. (**C**) ELISA of IL1β levels in clusterin-KO primary HCs and KCs treated with Tg (1 μM, 24 h). *p < 0.05, ***p < 0.001. (**D**) Representative western blot analysis of NLRP3 and IL1β protein expression in lysates of clusterin-KO primary HCs and KCs treated with Tg

ER stress has been implicated in liver injury and NLRP3 inflammasome activation. Therefore, we examined the status of various ER stress markers in the liver of DDC diet-fed mice. Immunohistochemical staining showed that expression of ATF6 and CHOP was increased in clusterin-KO mice fed a DDC diet for 20 days (Fig. 5A, B). Protein expression of p-eIF2α, ATF6, and CHOP, which are ER stress markers, was also increased in these mice (Fig. 5C). To determine if NLRP3 inflammasomes are generated in response to ER stress in various types of liver cells, we induced ER stress in primary HCs and KCs. Induction of ER stress activated NLRP3 inflammasomes in both HCs and KCs (Fig. S3). Next, we isolated primary HCs and KCs from clusterin-KO mice to investigate the role of clusterin in ER stress-induced NLRP3 inflammasome activation. The ER stress inducer Tg increased mRNA expression of NLRP3, IL1β, tumor necrosis factor alpha (TNFα), and interleukin 6 (IL6) in HCs and KCs, and these increases were exacerbated in HCs and KCs isolated from clusterin-KO mice (Fig. 6A, B). In addition, Tg further increased IL1β secretion and protein expression of NLRP3 and IL1β in HCs and KCs isolated from clusterin-KO mice (Fig. 6C, D). These results suggest that clusterin deficiency promotes ER stress-induced NLRP3 inflammasome activation in both HCs and KCs.

### Clusterin inhibits ER stress-induced NLRP3 inflammasome activation

**Fig. 7:**
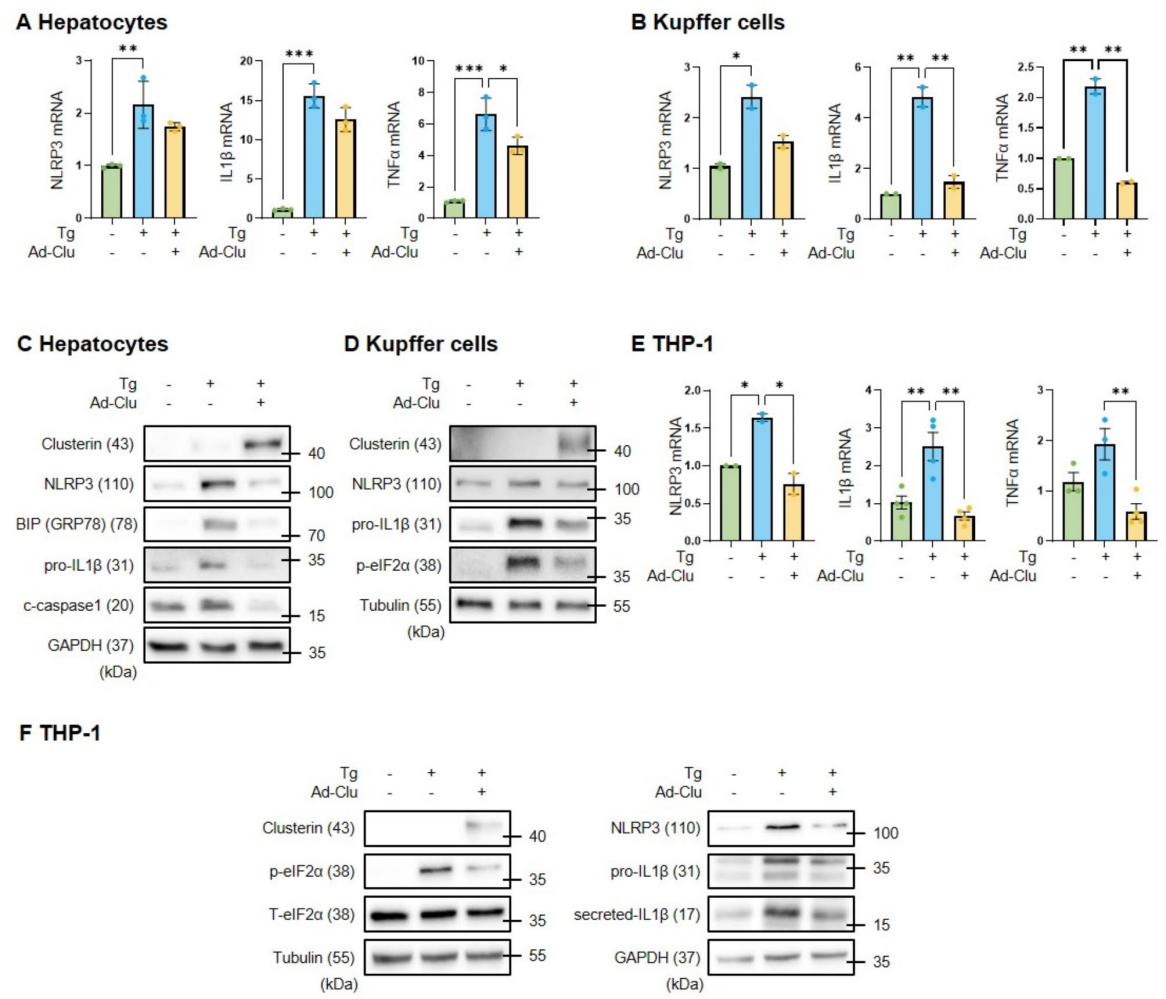
Clusterin attenuates ER stress-induced NLRP3 inflammasome activation. (**A**,** B**) Real-time RT-PCR analysis of the effects of clusterin on Tg-induced (1 µM, 24 h) mRNA expression of NLRP3, IL1β, and TNFα in primary HCs (**A**) and KCs (**B**). *p < 0.05, **p < 0.01, ***p < 0.001. (**C**,** D**) Western blot analysis of the effects of clusterin on Tg-induced protein expression of NLRP3 and IL1β in lysates of primary HCs (**C**) and KCs (**D**). (**E**,** F**) Real-time RT-PCR (**E**) and western blot (**F**) analyses of the effects of clusterin on Tg-induced NLRP3 and IL1β expression in THP-1 cells. *p < 0.05, **p < 0.01

To further evaluate the inhibitory effect of clusterin on ER stress-induced NLRP3 inflammasome activation, clusterin was overexpressed using a clusterin-containing adenovirus. Clusterin inhibited Tg-induced NLRP3, IL1β, and TNFα mRNA expression and p-eIF2α, NLRP3, and IL1β protein expression in KCs and HCs (Fig. 7A–D). Consistently, clusterin inhibited ER stress-induced NLRP3 inflammasome activation in the human monocyte cell line THP-1 (Fig. 7E, F). These results suggest that clusterin inhibits NLRP3 inflammasome activation by suppressing ER stress.

## Discussion

The present study demonstrated that clusterin deficiency promotes cholestatic liver disease by inducing ER stress and NLRP3 inflammasome activation and that clusterin inhibits ER stress-induced NLRP3 inflammasome activation.

Cholestasis refers to the accumulation of bile acids in the liver, leading to liver injury. DDC-fed animals are the most widely used animal models to study xenobiotic-induced liver injury. Feeding mice DDC induces cholestatic liver disease with perihepatic fibrosis and massive portal/periportal inflammatory infiltrate. Notably, cholestatic liver injury induced by a DDC diet is associated with an increased level of cytokeratin 19 (CK19), a marker of bile duct cells (Fig. S4). The increased level of CK19 indicates that proliferation of biliary epithelial cells continues, which is a key feature of biliary injury [22].

Clusterin plays an important role in the diagnosis and prognosis of various liver diseases, such as biliary atresia and hepatocellular carcinoma, and differentiation between liver and non-liver cancers. Upregulation of clusterin is a protective mechanism to prevent disease [23–26]. In our study, expression of clusterin was increased by feeding a DDC diet or treatment with bile acids, and was also increased in the livers of patients with PBC. In addition, clusterin-KO mice fed a DDC diet had elevated serum levels of ALT, AST, IL1β, and IL18, and more severe fibrosis, indicating that the liver was more vulnerable to cholestatic injury. We also reported that clusterin expression is increased in a BDL model [25], and liver damage was significantly more pronounced in clusterin-KO mice subjected to BDL, highlighting the important protective role of clusterin in cholestatic liver disease characterized by severe fibrosis.

The NLRP3 inflammasome is an important component of the innate immune system and is composed of the sensor molecules NLRP3 (NOD-, LRR-, and pyrin domain-containing protein 3), the adaptor protein ASC (apoptosis-associated speck-like protein containing CARD), and the effector molecule pro-caspase-1 [27]. NLRP3 inflammasome activation induces the secretion of cytokines such as IL1β [28, 29], which plays a role in inducing liver inflammation and fibrosis [28, 30]. Recent studies also highlighted the important role of another inflammasome cytokine, IL18, in liver damage. Elevated levels of IL18 have been observed in plasma and liver tissue of patients with cholestatic disorders, further supporting the involvement of the NLRP3 inflammasome in liver disease [31–33]. In our study, secretion of IL1β and IL18 was increased and NLRP3, ASC, and caspase-1 were upregulated in clusterin-KO mice fed a DDC diet. This upregulation triggered expression of GSDMD, which is associated with inflammatory cell death [34]. Moreover, the level of CHOP, which mediates ER stress-induced cell death, was elevated in clusterin-KO mice fed a DDC diet. These findings suggest there is a complex relationship between clusterin deficiency, NLRP3 inflammasome activity, and ER stress, which contributes to the exacerbation of liver injury. The ER stress response is associated with several metabolic and liver diseases, including fatty liver disease and cholestatic liver disease [5, 35]. It has also been reported that ER stress activates NLRP3 inflammasomes in HCs, leading to liver injury, inducing fever and cell death, and contributing to non-alcoholic steatohepatitis [10, 36]. Our findings clearly show that clusterin deficiency significantly upregulates ER stress and NLRP3 inflammasome activation upon cholestatic liver injury. Activation of NLRP3 inflammasomes induced by ER stress was enhanced in clusterin-KO HCs. These results suggest that clusterin deficiency also plays a role in promoting fibrosis and liver injury.

To directly test the regulatory role of clusterin in these enhanced stress responses, we performed additional experiments in which clusterin was overexpressed in HCs isolated from clusterin-KO mice. Clusterin overexpression significantly attenuated the Tg-induced increases of the NLRP3 and IL1β levels in clusterin-KO HCs, suggesting that clusterin mitigates inflammasome activation under stress conditions (Fig. S5A). In addition, treatment of HCs isolated from clusterin-KO mice with MCC950, a specific inhibitor of the NLRP3 inflammasome, also attenuated the Tg-induced increases of NLRP3 and IL1β (Fig. S5B). These results provide compelling evidence that decreasing NLRP3 activity by upregulating clusterin can effectively reduce the inflammatory response in HCs and confirm the therapeutic potential of modulating this pathway in liver diseases associated with increased inflammasome activity.

Phorbol 12-myristate 13-acetate-treated THP-1 macrophages release a variety of inflammatory mediators and serve as a cellular model of inflammatory diseases. THP-1 cells also play an important role in liver inflammation in response to a variety of stimuli. In chronic hepatitis C [37], THP-1 macrophages contribute to liver inflammation and disease progression. Similarly, in obesity-associated non-alcoholic fatty liver disease [38], inflammation induced by THP-1 macrophages plays a crucial role in liver injury and fibrosis, highlighting the importance of these cells in the mechanisms underlying liver inflammation. Clusterin inhibits ER stress-induced NLRP3 inflammasome activation in THP-1 cells, demonstrating that its anti-inflammatory effects are not limited to HCs but extend to other cell types involved in chronic inflammatory diseases. This highlights the potential of modulating clusterin as a therapeutic strategy targeting multiple components of the inflammatory pathway, including macrophages. Further studies of clusterin in biliary epithelial cells (cholangiocytes) are needed to fully understand its effects on bile duct and liver injury.

HSCs are crucial for liver fibrosis, primarily through their activation and transdifferentiation from quiescent cells into fibrotic myofibroblasts, which significantly contribute to the initiation and development of liver fibrosis [39, 40]. HCs, the main parenchymal cells of the liver, along with HSCs, drive the fibrotic response. Additionally, as part of the immune system, KCs also play a role in promoting liver fibrosis. Activation of NLRP3 inflammasomes in both HCs and KCs further stimulates activation of HSCs, leading to fibrosis and liver damage. Therefore, NLRP3 inhibitors can be used to prevent liver injury [41]. In this study, primary HSCs of clusterin-KO mice that were cultured for 7 days after isolation displayed higher levels of αSMA, NLRP3, and IL1β than those isolated from WT mice. Additionally, clusterin inhibited expression of NLRP3 and IL1β, as well as collagen and αSMA (Fig. S6A, B). In addition, clusterin inhibited Tg-induced mRNA expression of IL1β and protein expression of NLRP3, collagen, and αSMA in LX2 human HSCs (Fig. S6C, D). We previously demonstrated that clusterin inhibits liver fibrosis. This study provides further evidence that clusterin effectively reduces liver fibrosis under various experimental conditions.

## Conclusions

In conclusion, this study shows that upregulation of clusterin in the cholestatic liver may be protective. The absence of clusterin exacerbates ER stress and NLRP3 inflammasome activation in mice fed a DDC diet. Conversely, overexpression of clusterin suppresses ER stress and NLRP3 inflammasome activation. Therefore, clusterin deficiency is linked to an increased inflammatory response in the liver, which is associated with upregulation of ER stress.

## Electronic supplementary material

Below is the link to the electronic supplementary material.

Supplementary Material 1

## Data Availability

Data will be made available on request.

## Abbreviations

ALT: Alanine aminotransferase
AST: Aspartate aminotransferase
BDL: Bile duct ligation
CA: Cholic acid
CDCA: Chenodeoxycholic acid
CK19: Cytokeratin 19
ELISA: Enzyme-linked immunosorbent assay
ER: Endoplasmic reticulum
FBS: Fetal bovine serum
GBSS: Gey’s Balanced Salt Solution
GSDMD: Gasdermin D
HC: Hepatocyte
HSC: Hepatic stellate cell
IL1β: Interleukin 1 beta
IL6: Interleukin 6
IL18: Interleukin 18
KC: Kupffer cell
KO: Knockout
PBC: Primary biliary cholangitis
SEM: Standard error of mean
TCA: Taurocholic acid
Tg: Thapsigargin
TNFα: Tumor necrosis factor alpha
WT: Wild-type

## References

[1] Allen K, Jaeschke H, Copple BL. Bile acids induce inflammatory genes in hepatocytes: A novel mechanism of inflammation during obstructive cholestasis. Am J Pathol. 2011;178(1):175–86. 21224055 10.1016/j.ajpath.2010.11.026 PMC3070591

[2] Cai S-Y, Boyer JL. The role of inflammation in the mechanisms of bile Acid-Induced liver damage. Dig Dis. 2017;35(3):232–4. 28249287 10.1159/000450916 PMC6051694

[3] Cai SY, Ouyang X, Chen Y, Soroka CJ, Wang J, Mennone A et al. Bile acids initiate cholestatic liver injury by triggering a hepatocyte-specific inflammatory response. J Clin Invest. 2017;2(5). 10.1172/jci.insight.90780 PMC5333973 28289714

[4] Kullak-Ublick GA, Meier PJ. Mechanisms of cholestasis. Clin Liver Dis. 2000;4(2):357–85. 11232196 10.1016/s1089-3261(05)70114-8

[5] Malhi H, Kaufman RJ. Endoplasmic reticulum stress in liver disease. J Hepatol. 2011;54(4):795–809. 21145844 10.1016/j.jhep.2010.11.005 PMC3375108

[6] Henkel AS, LeCuyer B, Olivares S, Green RM. Endoplasmic reticulum stress regulates hepatic bile acid metabolism in mice. Cell Mol Gastroenterol Hepatol. 2017;3(2):261–71. 28275692 10.1016/j.jcmgh.2016.11.006 PMC5331781

[7] Zhu J, Wang R, Xu T, Zhang S, Zhao Y, Li Z, et al. Salvianolic acid A attenuates Endoplasmic reticulum stress and protects against Cholestasis-Induced liver fibrosis via the SIRT1/HSF1 pathway. Front Pharmacol. 2018;9:1277. 30455644 10.3389/fphar.2018.01277 PMC6230567

[8] Liu X, Green RM. Endoplasmic reticulum stress and liver diseases. Liver Res. 2019;3(1):55–64. 32670671 10.1016/j.livres.2019.01.002 PMC7363397

[9] Lv S, Li X, Wang H. The role of the effects of Endoplasmic reticulum stress on NLRP3 inflammasome in diabetes. Front Cell Dev Biology. 2021;9. 10.3389/fcell.2021.663528 PMC8079978 33937267

[10] Lu X, Huang H, Fu X, Chen C, Liu H, Wang H et al. The role of Endoplasmic reticulum stress and NLRP3 inflammasome in liver disorders. Int J Mol Sci. 2022;23(7). 10.3390/ijms23073528 PMC8998408 35408890

[11] Jo E-K, Kim JK, Shin D-M, Sasakawa C. Molecular mechanisms regulating NLRP3 inflammasome activation. Cell Mol Immunol. 2016;13(2):148–59. 26549800 10.1038/cmi.2015.95 PMC4786634

[12] de Carvalho Ribeiro M, Szabo G. Role of the inflammasome in liver disease. Annu Rev Pathol. 2022;17:345–65. 34752711 10.1146/annurev-pathmechdis-032521-102529 PMC10501045

[13] Szabo G, Csak T. Inflammasomes in liver diseases. J Hepatol. 2012;57(3):642–54. 22634126 10.1016/j.jhep.2012.03.035

[14] Koyama Y, Brenner DA. Liver inflammation and fibrosis. J Clin Investig. 2017;127(1):55–64. 28045404 10.1172/JCI88881 PMC5199698

[15] Jones SE, Jomary C, Clusterin. Int J Biochem Cell Biol. 2002;34(5):427–31. 11906815 10.1016/s1357-2725(01)00155-8

[16] Rosenberg ME, Silkensen J. Clusterin: physiologic and pathophysiologic considerations. Int J Biochem Cell Biol. 1995;27(7):633–45. 7648419 10.1016/1357-2725(95)00027-m

[17] Shim YJ, Kang BH, Jeon HS, Park IS, Lee KU, Lee IK, et al. Clusterin induces matrix metalloproteinase-9 expression via ERK1/2 and PI3K/Akt/NF-κB pathways in monocytes/macrophages. J Leukoc Biol. 2011;90(4):761–9. 21742938 10.1189/jlb.0311110

[18] Kang BH, Shim YJ, Tae YK, Song JA, Choi BK, Park IS, et al. Clusterin stimulates the chemotactic migration of macrophages through a pertussis toxin sensitive G-protein-coupled receptor and Gβγ-dependent pathways. Biochem Biophys Res Commun. 2014;445(3):645–50. 24569077 10.1016/j.bbrc.2014.02.071

[19] Shim YJ, Kang BH, Choi BK, Park IS, Min BH. Clusterin induces the secretion of TNF-α and the chemotactic migration of macrophages. Biochem Biophys Res Commun. 2012;422(1):200–5. 22575505 10.1016/j.bbrc.2012.04.162

[20] Liu G, Zhang H, Hao F, Hao J, Pan L, Zhao Q et al. Clusterin reduces cold Ischemia-Reperfusion injury in heart transplantation through regulation of NF-kB signaling and Bax/Bcl-xL expression. Cellular physiology and biochemistry: international journal of experimental cellular physiology, biochemistry, and Pharmacology. 2018;45(3):1003–12. 10.1159/000487295 29428944

[21] Li Y, Song YQ, Zhang Y, Liu T, Qin Q. Over-expression of Apolipoprotein J inhibits cholesterol Crystal-Induced inflammatory responses via suppressing NLRP3 inflammasome activation in THP-1 macrophages. Folia Biol (Praha). 2021;67(5–6):183–90. 35439851 10.14712/fb2021067050183

[22] Pose E, Sancho-Bru P, Coll M. 3,5-Diethoxycarbonyl-1,4-Dihydrocollidine diet: A rodent model in cholestasis research. Methods Mol Biol. 2019;1981:249–57. 31016659 10.1007/978-1-4939-9420-5_16

[23] Aigelsreiter A, Janig E, Sostaric J, Pichler M, Unterthor D, Halasz J, et al. Clusterin expression in cholestasis, hepatocellular carcinoma and liver fibrosis. Histopathology. 2009;54(5):561–70. 19413638 10.1111/j.1365-2559.2009.03258.x

[24] Zhou W, Guan Q, Kwan CCH, Chen H, Gleave ME, Nguan CYC, et al. Loss of clusterin expression worsens renal ischemia-reperfusion injury. Am J Physiology-Renal Physiol. 2009;298(3):F568–78. 10.1152/ajprenal.00399.2009 20007348

[25] Seo HY, Lee SH, Lee JH, Kang YN, Choi YK, Hwang JS et al. Clusterin attenuates hepatic fibrosis by inhibiting hepatic stellate cell activation and downregulating the Smad3 signaling pathway. Cells. 2019;8(11). 10.3390/cells8111442 PMC6912488 31739636

[26] Park J-S, Lee W-K, Kim HS, Seo JA, Kim D-H, Han HC, et al. Clusterin overexpression protects against Western diet-induced obesity and NAFLD. Sci Rep. 2020;10(1):17484. 33060605 10.1038/s41598-020-73927-y PMC7562726

[27] Tschopp J, Schroder K. NLRP3 inflammasome activation: the convergence of multiple signalling pathways on ROS production? Nat Rev Immunol. 2010;10(3):210–5. 20168318 10.1038/nri2725

[28] Latz E, Xiao TS, Stutz A. Activation and regulation of the inflammasomes. Nat Rev Immunol. 2013;13(6):397–411. 23702978 10.1038/nri3452 PMC3807999

[29] Gan C, Cai Q, Tang C, Gao J. Inflammasomes and pyroptosis of liver cells in liver fibrosis. Front Immunol. 2022;13:896473. 35707547 10.3389/fimmu.2022.896473 PMC9189314

[30] Kaufmann B, Leszczynska A, Reca A, Booshehri LM, Onyuru J, Tan Z, et al. NLRP3 activation in neutrophils induces lethal autoinflammation, liver inflammation, and fibrosis. EMBO Rep. 2022;23(11):e54446. 36194627 10.15252/embr.202154446 PMC9638850

[31] Yang L, Mizuochi T, Shivakumar P, Mourya R, Luo Z, Gutta S, et al. Regulation of epithelial injury and bile duct obstruction by NLRP3, IL-1R1 in experimental biliary Atresia. J Hepatol. 2018;69(5):1136–44. 29886157 10.1016/j.jhep.2018.05.038 PMC6314850

[32] El-Sayed IH, El kady IM, Badra GA. The effect of endoscopic retrograde cholangiopancreatography on the serum IL-18 and erythrocytes antioxidative capacity in biliary obstructive jaundice. Clin Chim Acta. 2003;336(1–2):123–8. 14500044 10.1016/s0009-8981(03)00336-x

[33] Maroni L, Agostinelli L, Saccomanno S, Pinto C, Giordano DM, Rychlicki C, et al. Nlrp3 activation induces Il-18 synthesis and affects the epithelial barrier function in reactive cholangiocytes. Am J Pathol. 2017;187(2):366–76. 27912077 10.1016/j.ajpath.2016.10.010

[34] Shi J, Zhao Y, Wang K, Shi X, Wang Y, Huang H, et al. Cleavage of GSDMD by inflammatory caspases determines pyroptotic cell death. Nature. 2015;526(7575):660–5. 26375003 10.1038/nature15514

[35] Dara L, Ji C, Kaplowitz N. The contribution of Endoplasmic reticulum stress to liver diseases. Hepatology (Baltimore MD). 2011;53(5):1752–63. 21384408 10.1002/hep.24279 PMC3082587

[36] Lebeaupin C, Proics E, de Bieville CHD, Rousseau D, Bonnafous S, Patouraux S, et al. ER stress induces NLRP3 inflammasome activation and hepatocyte death. Cell Death Dis. 2015;6(9):e1879–e. 26355342 10.1038/cddis.2015.248 PMC4650444

[37] Negash AA, Ramos HJ, Crochet N, Lau DT, Doehle B, Papic N, et al. IL-1β production through the NLRP3 inflammasome by hepatic macrophages links hepatitis C virus infection with liver inflammation and disease. PLoS Pathog. 2013;9(4):e1003330. 23633957 10.1371/journal.ppat.1003330 PMC3635973

[38] Ekinci I, Dumur S, Uzun H, Anataca G, Yalcinkaya I, Buyukkaba M, et al. Thrombospondin 1 and nuclear factor kappa B signaling pathways in Non-alcoholic fatty liver disease. J Gastrointestin Liver Dis. 2022;31(3):309–16. 36112712 10.15403/jgld-4390

[39] Tsuchida T, Friedman SL. Mechanisms of hepatic stellate cell activation. Nat Reviews Gastroenterol Hepatol. 2017;14(7):397–411. 10.1038/nrgastro.2017.38 28487545

[40] Charan HV, Dwivedi DK, Khan S, Jena G. Mechanisms of NLRP3 inflammasome-mediated hepatic stellate cell activation: therapeutic potential for liver fibrosis. Genes Dis. 2023;10(2):480–94. 37223529 10.1016/j.gendis.2021.12.006 PMC10201559

[41] Wree A, Eguchi A, McGeough MD, Pena CA, Johnson CD, Canbay A, et al. NLRP3 inflammasome activation results in hepatocyte pyroptosis, liver inflammation, and fibrosis in mice. Hepatology (Baltimore MD). 2014;59(3):898–910. 23813842 10.1002/hep.26592 PMC4008151

