# Supplementary material for: Clusterin deficiency exacerbates cholestatic liver disease through ER stress and NLRP3 inflammasome activation
Source: Cell Biosci. 2025 Mar 15;15:36. doi: 10.1186/s13578-025-01376-z (PMC11909925; doi:10.1186/s13578-025-01376-z)

**SUPPLEMENTARY DATA**

**
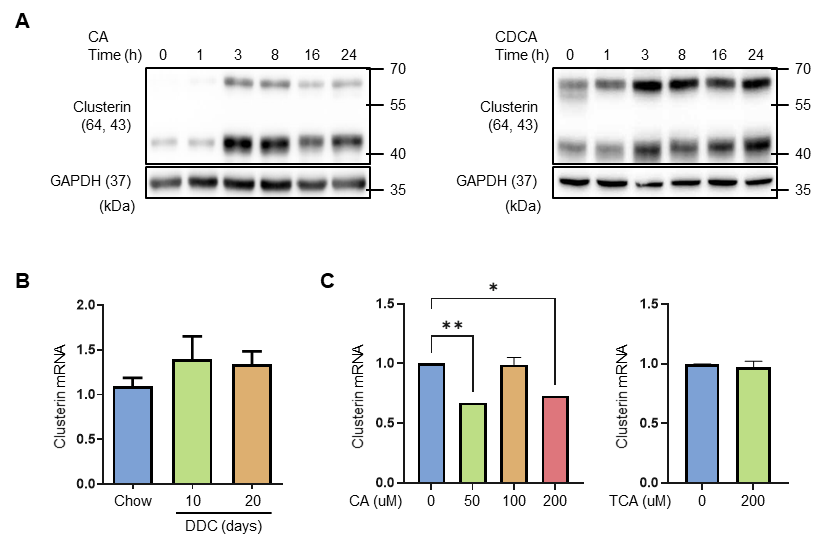
**

**Fig. S1** (**A**) Representative western blot analysis of clusterin expression in primary HCs treated with CA and CDCA. (**B, C**) Representative real-time RT-PCR analysis of clusterin mRNA expression in liver tissues of C57BL/6 mice fed a DDC diet (B) and primary HCs treated with CA and TCA (C). *p < 0.05, **p < 0.01.


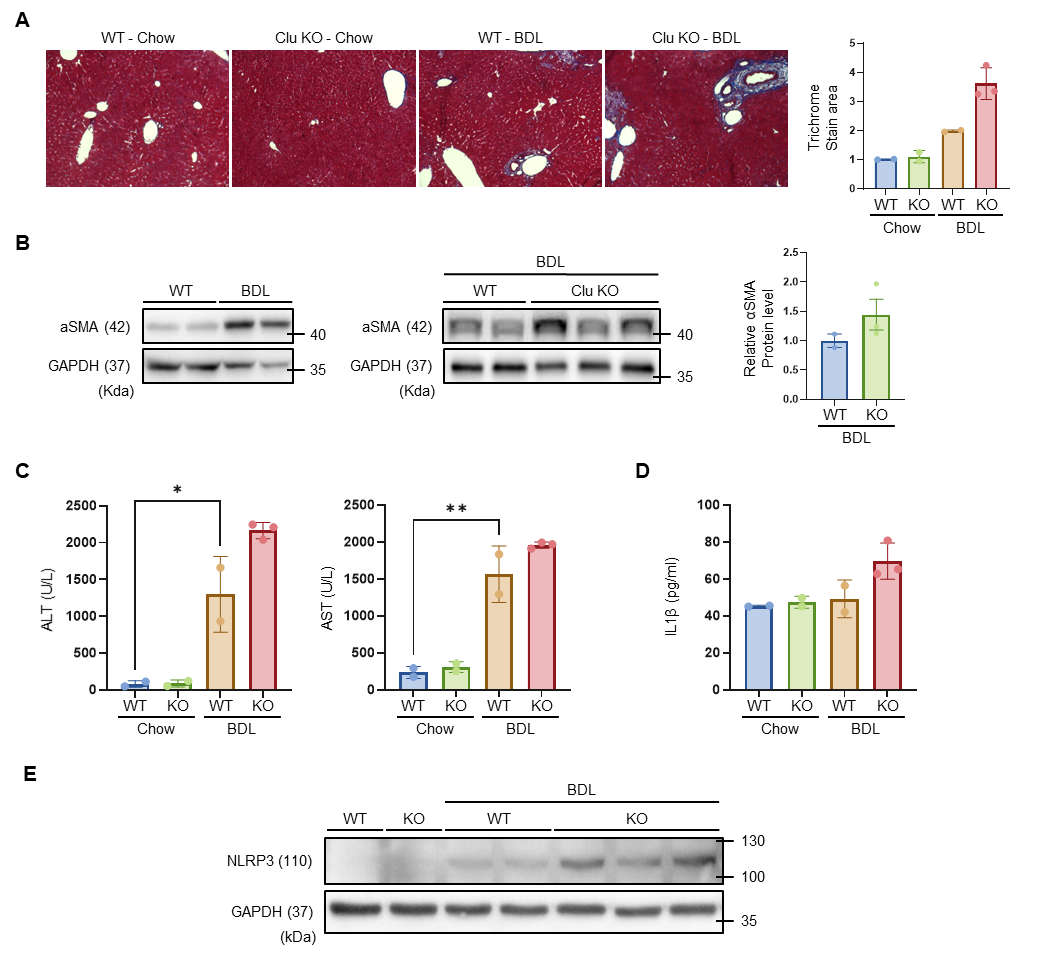


**Fig. S2** (**A**) Representative trichrome staining of liver sections from WT and clusterin-KO mice subjected to BDL. (**B**) Representative western blot analysis of αSMA expression in WT and clusterin-KO mice subjected to BDL. (**C**) ELISAs of serum ALT and AST levels in WT and clusterin-KO mice subjected to BDL. (**D**) An ELISA of serum IL1β levels in WT and clusterin-KO mice subjected to BDL. (**E**) Representative western blot analysis of NLRP3 expression in WT and clusterin-KO mice subjected to BDL. *p < 0.05, **p < 0.01.


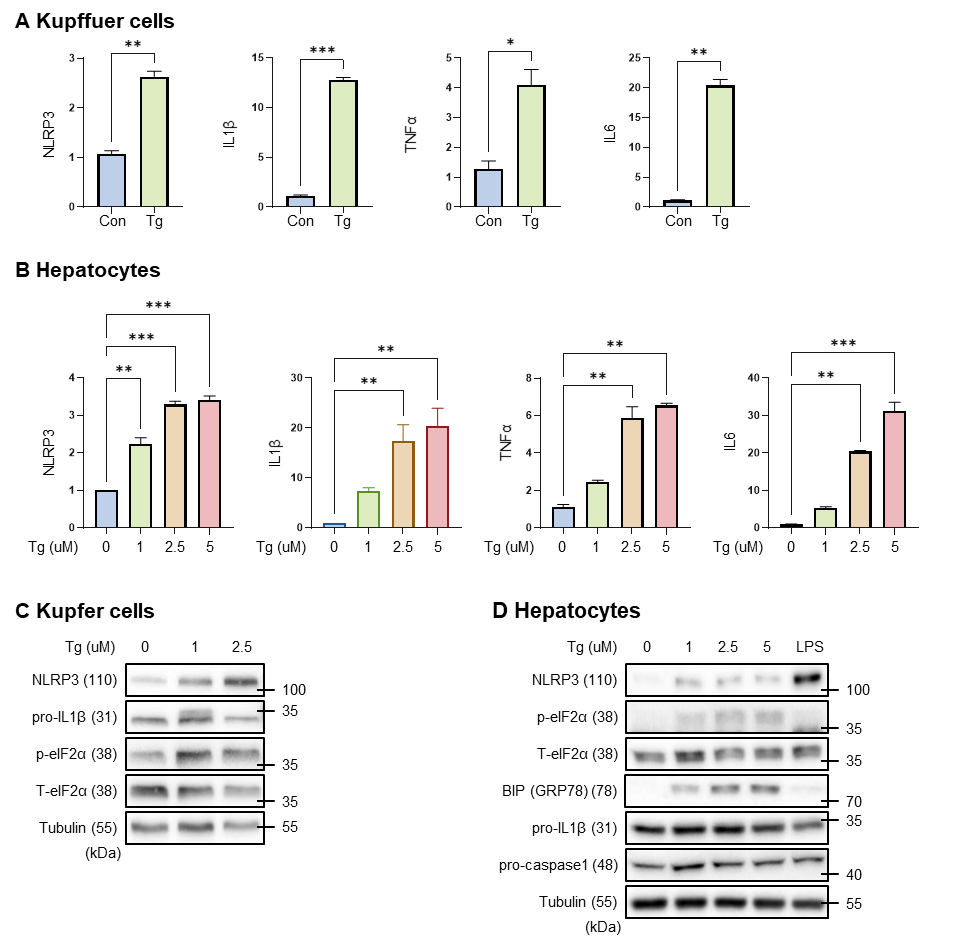


**Fig. S3** (**A, B**) Representative real-time RT-PCR analysis of NLRP3, IL1β, TNFα, and IL6 mRNA levels in KCs (A) and HCs (B) treated with Tg. *p < 0.05, **p < 0.01, ***p < 0.001. (**C, D**) Representative western blot analysis of NLRP3, IL1β, eIF2α, and caspase 1 protein levels in KCs (C) and HCs (D) treated with Tg.


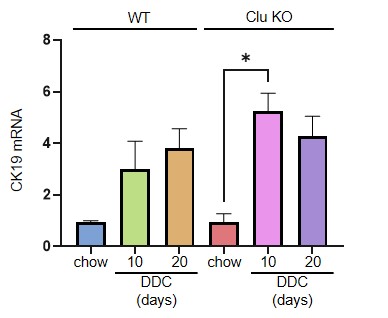


**Fig. S4** Representative real-time RT-PCR analysis of CK19 mRNA expression in liver tissues of WT and clusterin-KO mice fed a chow or DDC diet. *p < 0.05.

**
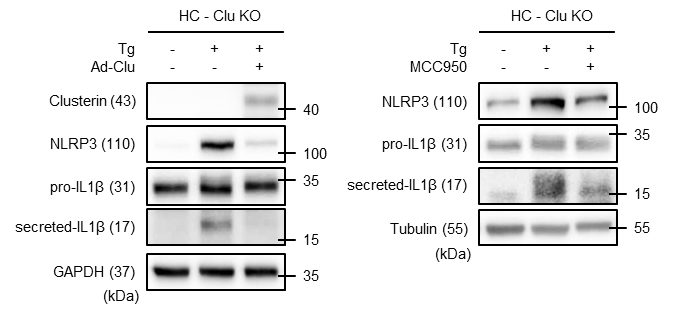
**

**Figure S5** (A) Representative western blot analysis of the effects of clusterin on Tg-induced expression of NLRP3 and IL1β in Clu-KO primary hepatocytes. (B) Representative western blot analysis of the effects of MCC950 on Tg-induced expression of NLRP3 and IL1β in Clu-KO primary hepatocytes.


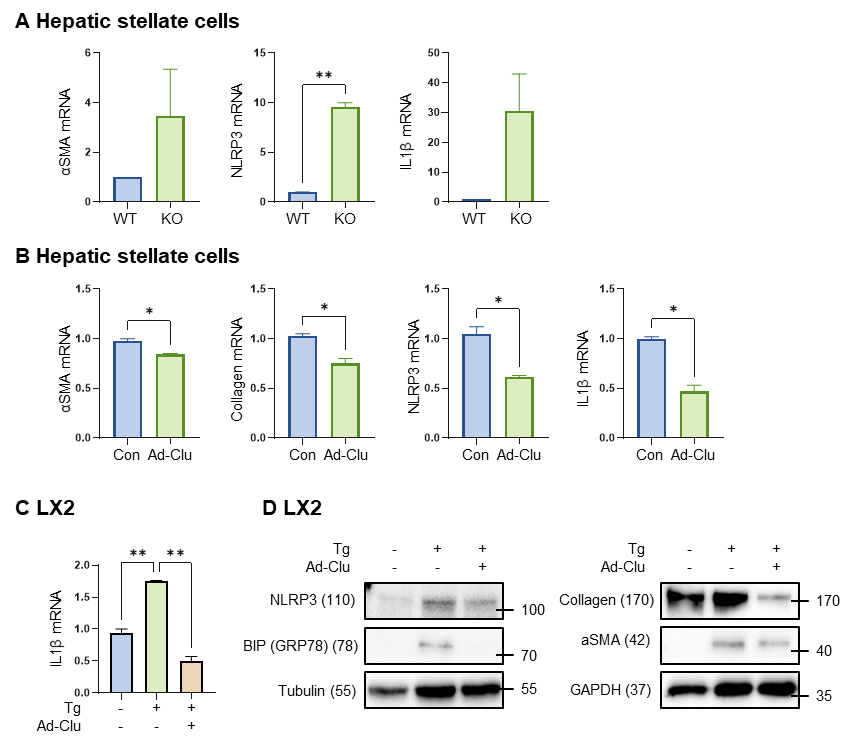


**Figure S6** (**A**) Representative real-time RT-PCR analysis of αSMA, NLRP3, and IL1β mRNA levels in WT and clusterin-KO primary HSCs. **p < 0.01. (**B**) Real-time RT-PCR analysis of the effects of clusterin overexpression on mRNA expression of αSMA, collagen, NLRP3, and IL1β in primary HSCs. *p < 0.05. (**C, D**) Real-time RT-PCR (C) and western blot (D) analyses of the effects of clusterin overexpression on Tg-induced IL1β, NLRP3, BiP, collagen, and αSMA expression in LX2 cells. **p < 0.01.


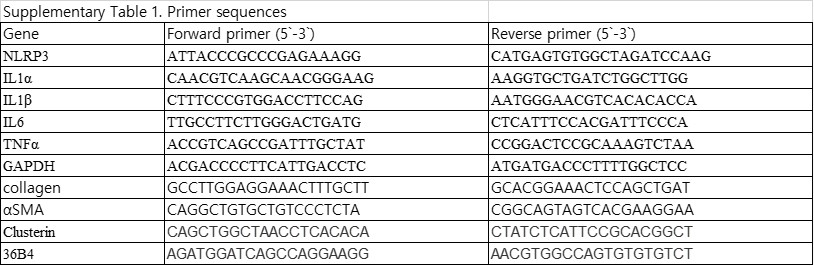

Supplement: Supplementary file 1 — Supplementary Material 1 [file 13578_2025_1376_MOESM1_ESM.docx]
